# Analysis of velocity- and power-load relationships of the free-weight back-squat and hexagonal bar deadlift exercises

**DOI:** 10.5114/biolsport.2023.112966

**Published:** 2022-02-18

**Authors:** Petrus Gantois, Fabiano de Souza Fonseca, Fábio Yuzo Nakamura, Leonardo de Sousa Fortes, Jaime Fernandez-Fernandez, Gilmário Ricarte Batista

**Affiliations:** 1 Associate Graduate Program in Physical Education UPE/UFPB. João Pessoa-PB, Brazil; 2 Federal Rural University of Pernambuco (UFRPE), Recife-PE, Brazil; 3 Graduate Program in Physical Education UFPE, Recife-PE, Brazil; 4 Research Center in Sports Sciences, Health Sciences and Human Development (CIDESD), University of Maia (ISMAI), Maia, Portugal; 5 Department of Physical Activity and Sport Sciences, Universidad de León, León, Spain

**Keywords:** Kinematic, Resistance exercise, Velocity-based training, Power output, Strength testing

## Abstract

The aim of this study was to analyse the load-velocity and load-power relationships in the free-weight back-squat (BSQ) and hexagonal bar deadlift (HBD) exercises. Twenty-five (n = 25) resistance-trained men (age = 23.7 ± 2.8 years) performed a progressive load test at maximal intended velocity to determine their BSQ and HBD one-repetition maximum (1RM). Mean propulsive velocity (MPV) during the concentric phase of the lift was recorded through a linear encoder. Load-velocity and load-power relationships were analysed by fitting linear regression and the second-order polynomial, respectively, to the data. Maximum strength (1RM), MPV (30–80% 1RM), and power output (30–90% 1RM) were higher for HBD compared to BSQ exercise (p < 0.05). A very strong relationship between MPV and relative intensity was found for both BSQ (R^2^ = 0.963) and HBD (R^2^ = 0.967) exercises. The load that maximizes power output (P_max_) was 64.6 ± 2.9% (BSQ) and 59.6 ± 1.1% (HBD) 1RM. There was a range of loads at which power output was not different than P_max_ (BSQ: 40–80% 1RM; HBD: 50–70% 1RM). In conclusion, the load-velocity and load-power relationships might assist strength and conditioning coaches to monitor and prescribe exercise intensity in the BSQ and HBD exercises using the velocity-based training approach.

## INTRODUCTION

Exercise intensity is one of the most important variables in resistance training (RT) for increasing muscular strength [1]. The one-repetition maximum (1RM) and the maximum number of repetitions (RMs) are among the main reference parameters used to determine exercise load [1, 2]. Although the direct assessments of 1RM and RMs are considered valid and effective approaches for prescribing exercise loads [3, 4], their use is often associated with some drawbacks which may limit their implementation (e.g., time-consuming and fatiguing procedures and 1RM daily fluctuations) [2, 3, 5].

Velocity-based training (VBT) has been proposed as a feasible alternative to the traditional 1RM-based approach to support the prescription and adjustment of the exercise load in a variety of resistance exercises due to the very strong and stable load-velocity relationships (R^2^ range: 0.94 to 0.98) [2, 6–8]. The use of general load-velocity relationship equations enables strength and conditioning coaches to easily determine which relative load (%1RM) is being lifted from the bar velocity recorded during a single repetition performed at maximum effort [2]. These data highlight the need for further investigation of the entire load-velocity relationship in the most common resistance exercises employed in RT programmes to successfully implement the velocity-based approach.

The back-squat (BSQ) and deadlift exercises and their variations are among the most common resistance exercises used to enhance the strength of knee extensors and flexor muscles [9]. Different exercise modes (Smith machine vs. free-weight, range of motion, and equipment design) produce distinct kinematic outputs and load-velocity relationships [10–13]. For example, previous studies showed that modifying the squatting depth (i.e. full vs. half) resulted in different biomechanical patterns, force development, and kinematic outputs [6, 10]. The load-velocity relationship in the Smith machine BSQ and traditional deadlift (i.e., straight bar) exercises has already been described [8, 10, 14–17]. However, reports of the load-velocity and load-power relationship in variations of the back-squat (i.e. free-weight) and traditional deadlift (i.e., hexagonal bar) exercises are limited.

Despite the popularity of both free-weight BSQ and hexagonal deadlift (HBD) exercises among strength and conditioning coaches, to the best of the authors’ knowledge, no study has compared the kinematic outputs of these exercises. From a mechanical perspective, shifting the load position can alter the kinematics and kinetics in lifting [18–20]. For example, the HBD makes it possible to keep the load closer to the individual’s body, reducing the amount of torso inclination and horizontal displacement of the bar, which may provide favourable vertical displacement over the BSQ exercise [18, 20, 21]. From a practical perspective, the resulting equations provided here would enable strength and conditioning coaches to incorporate the VBT paradigm for the free-weight BSQ and HBD exercises in their RT programmes.

Another important application of measuring bar velocity is to determine the range of loads able to maximize power output through the load-power relationship [10, 17], defined as the “optimum power zone” [22]. There is evidence supporting the effectiveness of training in the “optimum power zone” to enhance strength performance [22, 23]. Identifying this range of loads might be useful for strength and conditioning coaches to determine lower and upper limits of loads capable of producing high mechanical power output instead of using a single “optimal load” [22].

Given the above, the aims of this study were: (i) to determine the accuracy of movement velocity to estimate 1RM through the general and individual load-velocity relationship in the free-weight BSQ and HBD exercises; and (ii) to compare the load-velocity and load-power relationship of both exercises in resistance-trained males. Given the very strong load-velocity relationship previously reported [6, 16, 17], it was hypothesized that movement velocity would accurately predict 1RM for both exercises, but the load-velocity and load-power relationships would be exercise-dependent.

## MATERIALS AND METHODS

### Participants

Twenty-five (n = 25) resistance-trained men aged from 18 to 30 years (age = 23.7 ± 2.8 years; weight = 81.3 ± 8.4 kg; height = 1.77 ± 0.1 m) volunteered to participate in this cross-sectional study. All participants had experience in RT of 4.4 ± 1.3 years, with a training frequency of 2–5 RT sessions per week in the last 12 months, and were capable of performing both exercises with proper technique. All participants were free of muscular and joint injuries at the time of the study. The study was approved by the local Ethics Committee following the Declaration of Helsinki. All participants signed an informed consent form.

### Study design

Participants were required to attend the training facilities in three visits, with ˜48 h separating visits 1 and 2, and at least ˜1 week of rest between visits 2 and 3. The first visit involved the familiarization session (2 sets of 8 and 6 repetitions with 40 and 60 kg for both exercises), and visits 2 and 3 included the direct assessment of 1RM for back-squat and HBD exercises (randomized order) using a linear encoder for collecting mechanical output. Each visit was preceded by a standardized warm-up (10 min), which included dynamic stretching and joint mobility (3 min), light running (5 min), and a specific back-squat or HBD warm-up set for each exercise (1 set of 8 reps with 40 kg [BSQ] and 43 kg [HBD]), separated by 2 min of recovery. Participants were requested to attend each visit in a well-rested state (i.e., ≥ 8 hours sleep, to maintain their normal dietary habits, and to abstain from any physical exercise and beverages containing alcohol and caffeine ingestion in the 24 h before the sessions). Testing sessions were performed from 2:00 to 6:00 p.m., at the same time of day for each participant (± 1 hour).

### Testing procedures

After warming up, subjects rested for 2 min before starting the 1RM test. The initial load was set as 30 kg and 43 kg for the BSQ and HBD exercises, respectively. The load was increased by 20 kg until reaching a mean propulsive velocity (MPV) of 0.8 m · s^-1^ and 0.6 m · s^-1^ for BSQ and HBD, respectively, followed by increments of 10–5 kg (MPV = 0.8–0.5 m · s^-1^ and 0.6–0.4 m · s^-1^ for BSQ and HBD, respectively), and 5–1 kg up to 1RM. Three repetitions were performed for light (MPV > 0.8 m · s^-1^), two for medium (MPV = 0.5–0.8 m · s^-1^), and one for heavy (MPV < 0.5 m · s^-1^) loads, with an inter-set rest period of 2 min, 3 min, and 4 min, respectively. Each participant received real-time feedback of the bar velocity and verbal encouragement to exert their maximum effort at every repetition. The fastest MPV in each set was recorded for analysis.

The eccentric portion of the movement was executed in a continuous and controlled manner (2–3 s) with a momentary pause (˜1.5 s) between the eccentric and concentric phases to minimize the contribution of the rebound effect and provide more reliable data for both exercises [24]. The duration of the eccentric and isometric phases was paced by one of the investigators. This had been previously practised in the familiarization session. Ankle plantar flexion was allowed at the end of the movement for the light and moderate loads in both exercises, but without lifting the toes off the ground. If a repetition failed to meet these requirements, the set was discarded and repeated following a 3 min rest. This was visually supervised by one of the investigators examining the proper lifting technique during the test.

The BSQ exercise was performed as previously described [25]. Participants started from an upright position, with knees and hips fully extended, feet approximately shoulder-width apart and pointing slightly outward. The bar was placed in the upper portion of the trapezius muscle. This position was individually adjusted and replicated on every lift. From this position, they were required to flex their knees to ˜90° (determined by an elastic band placed parallel to the ground by a tripod), then fully extended their lower limbs at the end of lifting. The HBD exercise started with the bar on the floor with the high-handle grip following the previous recommendations [21]. The shoulder should be in line with the handles and hips lower than the shoulders. Stance position should be with feet approximately shoulder-width apart, both pointing slightly outward. From this position, they were instructed to lift the load and continue standing erect (full-arm-knee-hip extension) with their shoulder retracted for 1.5 s at the end of the lift. The bar was held on the ground for 1.5 s before each lift. No belts or straps were allowed [19].

### Measurement equipment and data recording

A linear position transducer (Speed4lifts, Madrid, Spain) was attached to the bar to measure the lifting kinematics. This system measures the cable displacement in response to changes in the bar position during the concentric phase at a sampling rate of 100 Hz [26]. The velocity parameter used in the present study was the MPV (i.e., the portion of the concentric phase in which the measured acceleration [α] is greater than the acceleration due to gravity [α > -9.81 m · s^-2^] [27]. MPV and the weight of the load lifted (bar + weight plate) were considered to calculate the mechanical power output (P) as follows [28]:


P=(Loadlifted⋅g)⋅MPV

where g is the gravitational acceleration (α), which is equal to 9.81 m · s^-2^.

The Speed4Lifts system has been demonstrated valid and reliable to record movement velocity for medium and heavy loads, with the absolute error below the acceptable maximum error criterion (< 5% of 1RM) [26, 29].

### Statistical analysis

We used standard descriptive statistics to calculate the mean, standard deviation (SD), percentage of coefficient of variation (%CV), standard error of the estimation (SEE), and confidence interval (95% CI) of the outcomes. Load-velocity and load-power relationships were analysed by fitting linear and second-order polynomial regression to all data points, respectively. The goodness of fit of the load-velocity and load-power relationships were analysed using the coefficient of determination (R^2^) and the 95% CI.

The accuracy of the general load-velocity relationship was obtained using the load closest to 80% 1RM and loads from 30 to 80% 1RM were used to estimate the 1RM from individual load-velocity relationships [16]. We used the minimal velocity threshold as the upper limit of the V1RM obtained from the general load-velocity relationship (i.e., BSQ [0.31 m · s^-1^] and HBD [0.30 m · s^-1^]) to estimate 1RM through the individual load-velocity relationship (Table 1). After determining the %1RM related to the selected load (˜80% 1RM) through the general equation, the following cross-multiplication was used to estimate the 1RM (30):

**TABLE 1:** Mean propulsive velocity attained at which relative intensity (%1RM) in the free half back-squat and hexagonal bar deadlift exercises (Mean and standard deviation).

Half Back-Squat				Hexagonal Bar Deadlift			
Load (%1RM)	MPV (m · s^-1^)	95% CI (m · s^-1^)	CV (%)	MPV (m · s^-1^)	95% CI (m · s^-1^)	CV (%)	P-Value
30	1.09 ± 0.07	1.06 to 1.12	6.60	1.29 ± 0.12	1.24 to 1.33	8.95	< 0.001
35	1.03 ± 0.07	1.00 to 1.06	6.54	1.21 ± 0.11	1.17 to 1.26	8.83	< 0.001
40	0.97 ± 0.06	0.95 to 1.00	6.47	1.14 ± 0.10	1.10 to 1.18	8.70	< 0.001
45	0.92 ± 0.06	0.90 to 0.94	6.40	1.07 ± 0.09	1.03 to 1.11	8.55	< 0.001
50	0.86 ± 0.05	0.84 to 0.89	6.33	1.00 ± 0.08	0.97 to 1.03	8.39	< 0.001
55	0.81 ± 0.05	0.79 to 0.83	6.26	0.93 ± 0.08	0.90 to 0.96	8.21	< 0.001
60	0.75 ± 0.05	0.73 to 0.77	6.20	0.86 ± 0.07	0.83 to 0.88	8.00	< 0.001
65	0.70 ± 0.04	0.68 to 0.71	6.15	0.78 ± 0.06	0.76 to 0.81	7.78	< 0.001
70	0.64 ± 0.04	0.62 to 0.66	6.11	0.71 ± 0.05	0.69 to 0.73	7.54	< 0.001
75	0.58 ± 0.04	0.57 to 0.60	6.12	0.64 ± 0.05	0.62 to 0.66	7.29	0.001
80	0.52 ± 0.03	0.52 to 0.54	6.19	0.57 ± 0.04	0.55 to 0.59	7.04	0.017
85	0.47 ± 0.03	0.46 to 0.48	6.37	0.50 ± 0.03	0.48 to 0.51	6.85	0.120
90	0.42 ± 0.03	0.40 to 0.43	6.73	0.43 ± 0.03	0.42 to 0.44	6.84	0.537
95	0.36 ± 0.03	0.35 to 0.37	7.40	0.36 ± 0.03	0.34 to 0.37	7.28	0.872
100	0.30 ± 0.03	0.29 to 0.31	8.57	0.29 ± 0.02	0.28 to 0.30	8.75	0.334

MPV = mean propulsive velocity; CI = confidence interval; CV = coefficient of variation.


1RM=Load(kg)×100/%1RM

We used a paired t-test to compare the average MPV values attained with each %1RM between exercises. The agreement between actual and predicted 1RM was assessed by intraclass correlation coefficient analysis, Bland-Altman plots and their 95% limits of agreement. A two-way (exercise × load) ANOVA was used to compare the mechanical power output at different loads (i.e., %1RM and percentage of body mass). Bonferroni’s post-hoc analysis was used to identify the significant differences. The analysis was performed in SPSS (IBM Corporation, New York, USA). The significance level was set at 5%.

## RESULTS

The 1RM for the 25 participants included in this study was 123.20 ± 13.63 kg (BSQ) and 149.00 ± 19.77 kg (HBD). The 1RM normalized per kg of body mass was 1.54 ± 0.24 (BSQ) and 1.86 ± 0.29 (HBD). The average number of attempts during the progressive test was 8.3 loads (range: 7–10) for the BSQ and 8.5 attempts (range: 7–10) for the HBD exercise.

### Load-velocity relationship

**FIG. 1:**
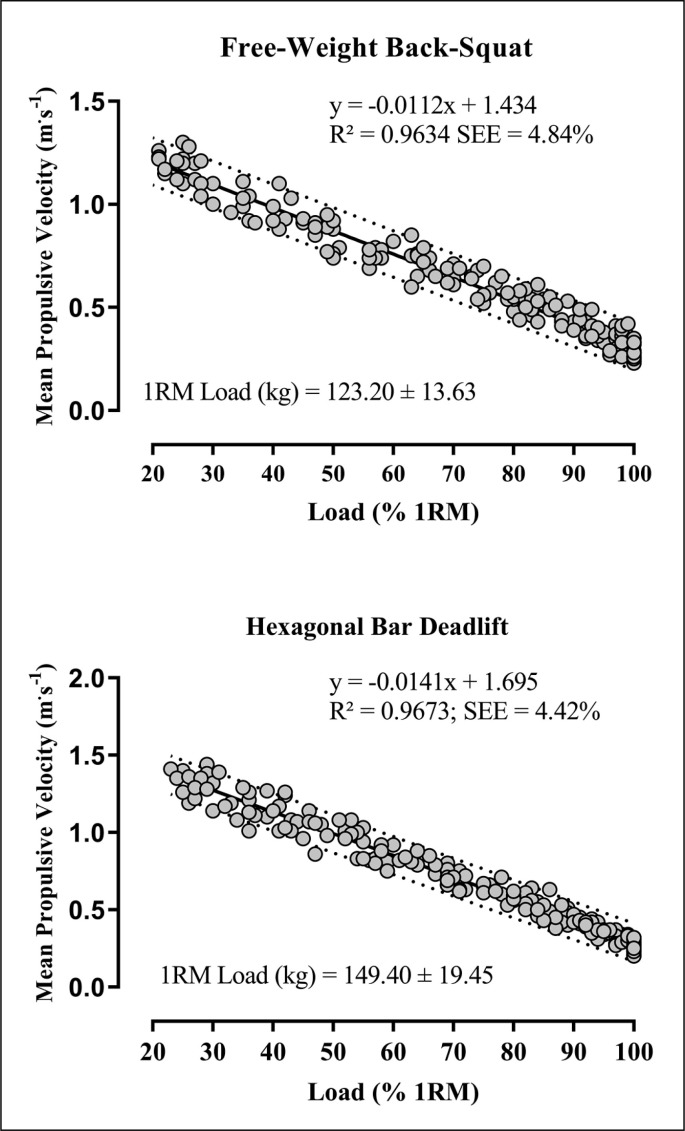
General linear load-velocity relationship for free half back-squat and hexagonal bar deadlift exercises. Solid line shows the fitted curve to the data, and the dotted lines indicate the predicted 95% CI.

Figure 1 shows the results of the linear regression, plotting MPV against each %1RM for both the BSQ and HBD exercises for all data points (BSQ: n = 210; and HBD: n = 211). A very strong load-velocity relationship was found for the BSQ (R^2^ = 0.963; SEE = 4.84%) and HBD (R^2^ = 0.967; SEE = 4.42%) exercises. The individual load-velocity relationship provided better adjustments for the BSQ (R^2^ = 0.983 ± 0.01) and HBD (R^2^ = 0.989 ± 0.01) exercises than the general equation.

**FIG. 2:**
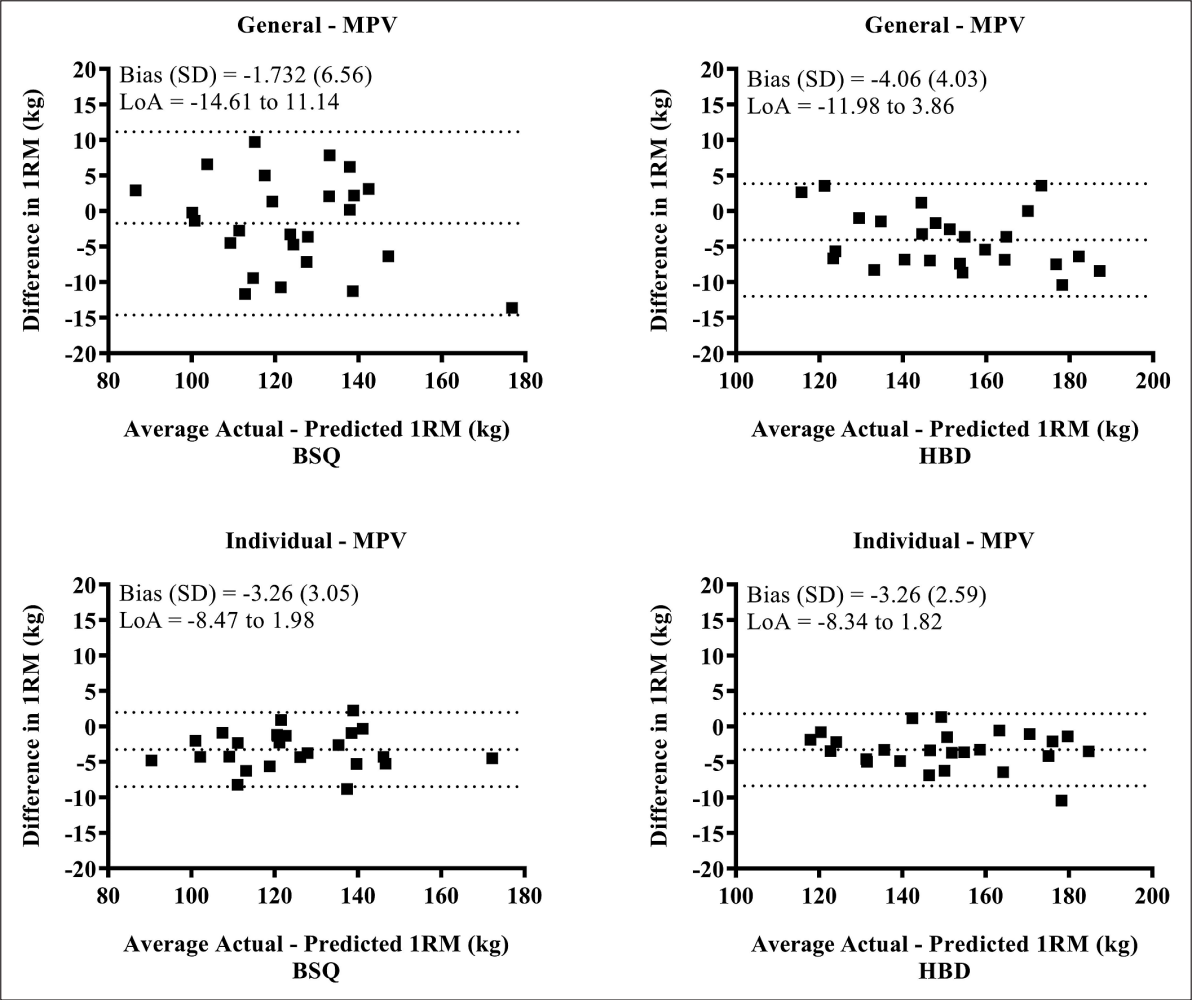
Bland-Altman plots between actual and predicted 1RM load obtained from the general and individualized load-velocity relationship for the free half back-squat and hexagonal bar deadlift exercises. BSQ = back-squat exercise; HBD = hexagonal bar deadlift; SD = standard deviation; LoA = limits of agreement.

Bland-Altman plots showed high agreement between actual and predicted 1RM for both the BSQ (ICC = 0.968; 95% CI = 0.928 to 0.986) and HBD (ICC = 0.981; 95% CI = 0.831 to 0.994) exercises, using general equations (Figure 2). The individual load-velocity relationship showed higher agreement than the general equation for both the BSQ (ICC = 0.987; 95% CI = 0.785 to 0.997) and HBD (ICC = 0.989; 95% CI = 0.810 to 0.997) exercises.

### Predicting relative load (%1RM) from MPV data

The following equations were obtained from the linear regression to estimate %1RM for both exercises:

*BSQ load (%1RM) = -85.638 MPV + 125.43 (R^2^ = 0.963; SEE = 4.84%)*

*HBD load (%1RM) = -68.648 MPV + 118.79 (R^2^ = 0.967; SEE = 4.42%)*

The average MPV and the between-subjects %CV attained with each %1RM obtained from the general load-velocity relationship from 30% onwards up to 1RM, in 5% increments, are displayed in Table 1. MPV attained with loads below 80% 1RM was significantly higher for the HBD (p < 0.05) than the BSQ exercise.

### Load-power relationships

**FIG. 3:**
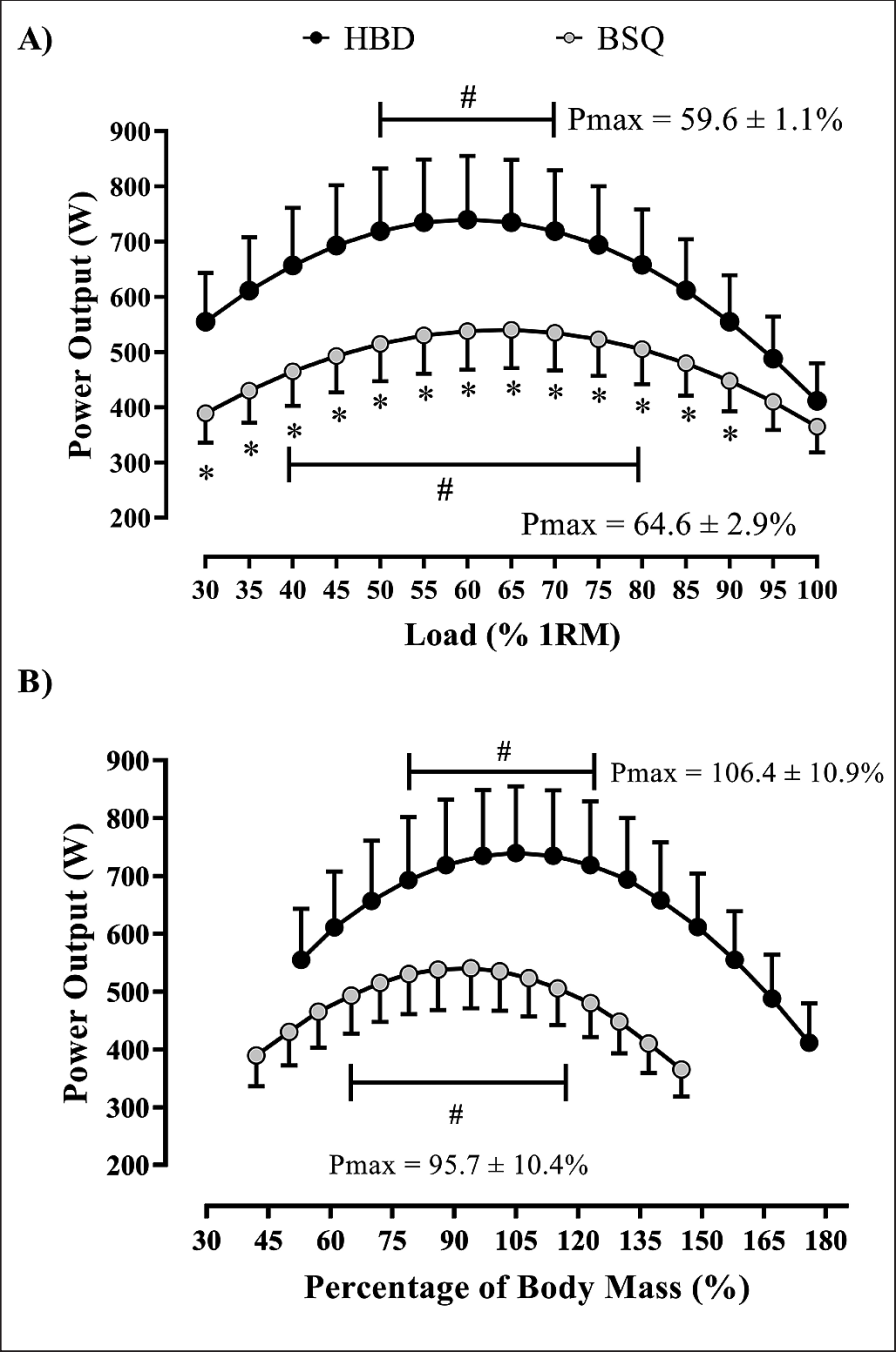
Load-power relationship for the free half back-squat and hexagonal bar deadlift exercises according to the %1RM (A) and percentage of body mass (B). HBD = hexagonal bar deadlift exercise; BSQ = back-squat exercise; open circle represents the Pmax = load at which power output is maximized; # = the range of loads at which the power output is not significantly different than Pmax; * = Statistically significant difference between exercises at the same relative load; percentage of body mass = relative load from 30% onwards until 100% of 1RM, in 5% increments.

The load which maximizes power output (P_max_) was 64.6 ± 2.9% (CV = 4.56%) and 59.6 ± 1.1% (CV = 1.83%) 1RM for the BSQ and HBD exercises, respectively (Figure 3). However, no significant differences were found for a range of %1RM for the BSQ (40–80% 1RM) and HBD (50–70% 1RM) exercises regarding P_max_ (p > 0.05). Higher power output was found for the HBD exercise than BSQ exercise (%1RM range: 30–90% 1RM; p < 0.05) (Figure 3a). Furthermore, the P_max_ relative to the percentage of body mass was 95.7 ± 10.4% (CV = 10.86%) and 106.4 ± 10.9% (CV = 10.25%) for BSQ and HBD, respectively. Nevertheless, no significant differences were found for a range of percentage of body mass for the BSQ (˜65–123%) and HBD (˜80–132%) exercises regarding P_max_ (p > 0.05) (Figure 3b).

## DISCUSSION

The main findings of the present study were as follows: (a) the load-velocity relationships in both the BSQ and HBD exercises were highly linear, which enables accurate load prescription from movement velocity; (b) HBD elicited higher kinematic outputs in a range of %1RM (< 90% 1RM) compared to BSQ exercise; (c) the loads that maximize the power output were ˜66% for the BSQ and ˜60% 1RM for the HBD. However, no power output differences were found in a wide range of %1RM related to the P_max_ for the BSQ (40–80% 1RM) and HBD (50–70% 1RM) exercises.

The very strong load-velocity relationships for both BSQ and HBD exercises (R^2^ > 0.96; SEE < 5% 1RM) enable strength and conditioning coaches to determine with great precision the 1RM from the MPV during a single repetition performed at maximum effort using submaximal loads on a daily basis. This statement is reinforced by the low %CV found for the MPV attained at each %1RM (< 10%) and the high agreement between actual and predicted 1RM (ICC > 0.96) in both exercises. The MPV difference between each 5% increment in %1RM was ˜0.06 m · s^-1^ (BSQ) and ˜0.07 m · s^-1^ (HBD). From a practical perspective, this means that changes in movement velocity of ± 0.06 m · s^-1^ (BSQ) and ± 0.07 m · s^-1^ (HBD) might represent a need to adjust the training load (± 5% 1RM).

The strong load-velocity relationship in the BSQ performed on the Smith machine [6, 10, 15] and the traditional deadlift [8, 16, 17, 31] has already been described, and the current study has extended that understanding to their variants (i.e., free-mode BSQ and HBD). Performing the BSQ using the Smith machine equipment limits the horizontal displacement of the bar in lifting, which might enhance the model prediction [13]. Nevertheless, our data showed that the velocity-based approach can also be used to estimate 1RM in the free-mode BSQ (R^2^ = 0.96). Likewise, to our knowledge, this is the first study to show that the bar velocity can estimate the 1RM in the HBD variant (R^2^ = 0.96). Given that the free-mode BSQ and HBD exercises are widely used in RT programmes to increase lower-body strength [9, 32], our results have important practical application by providing general equations to assist strength and conditioning coaches to incorporate the VBT paradigm in these exercises.

The individual load-velocity relationship showed a slightly better (but not relevant) adjustment of the load-velocity relationship (R^2^ > 0.98 versus R^2^ > 0.96, for the general equation). This is not surprising since the general equations do not consider some individuals’ characteristics that might affect the kinematic outputs in lifting (i.e., age, height, and limb lengths) [33, 34]. However, in practical terms, it is important to consider how meaningful these slight differences between general and individual load-velocity relationships are regarding training prescription or loading adjustment. For example, previous studies have already demonstrated the efficiency of the group-based equations to induce muscular strength and power adaptations [35, 36]. In a practical setting, strength and conditioning coaches seek to implement valid methods for prescribing the exercise load, but also simple and easy approaches to determine how their practitioners are responding to training. Hence, we suggest that the general equations provided here can be safely used to prescribe and adjust exercise load as a user-friendly and time-saving approach.

Supporting our second hypothesis, the relationship between %1RM and movement velocity was exercise-dependent (≤ 80% 1RM; p < 0.05). HBD exercise elicited higher mechanical power in a range of loads when compared to the BSQ exercise (30–90% 1RM; p < 0.05). Therefore, the applied force against light-moderate loads was greater during the HBD, since the lighter the %1RM was, the greater was the MPV difference between the two exercises. The difference in bar velocity found in our study might be explained by the advantageous lifting position of the external load when the HBD exercise is performed (i.e., upright torso position, reduced horizontal displacement of the bar, and less resistive torque) compared to the bar positioned on the shoulder (i.e., BSQ exercise) [18, 20, 21]. Therefore, it seems that the HBD can be prescribed to alter the movement pattern, muscular requirements, and kinematic outputs during RT programmes. Future studies are warranted to investigate how this kinematic advantage might translate into additional training adaptations.

Despite the clear difference in mechanical power between exercises, we observed that the loads related to P_max_ occurred at quite similar %1RM for both the BSQ (˜65% 1RM; MPV = ˜0.70 m · s^-1^) and HBD (˜60% 1RM; MPV = ˜0.86 m · s^-1^) exercises. However, loads in the range 40–80% (BSQ) and 50–70% 1RM (HBD) did not differ statistically concerning P_max_ (Figure 3). These data strongly support previous studies showing that mechanical power output is quite similar across a range of light-moderate loads in other resistance exercises such as the bench press (20–60% 1RM) [37], bench pull (20–70% 1RM) (37), traditional deadlift (40–80% 1RM) [17], and half-BSQ (25–85% 1RM) [10]. These findings raise some questions about how much attention has been given to determining a single “optimal load” [24, 37, 38]. This is supported by previous studies showing improvements in strength-power ability using a wide range of moderate (i.e. 55–70% 1RM) [39] to heavy loads (i.e. 70–85% 1RM) [40]. From a practical perspective, these data can be used for prescribing exercise load based on the individuals’ training goals (i.e., velocity- or force-biased).

Although there is a growing number of devices available to assess kinematics in resistance exercise, they may be not accessible to many RT practitioners. When such devices are unavailable, a possible alternative is prescribing exercise load based on the percentage of body mass [38]. When plotting the mechanical power output by individuals’ body mass percentage, our results showed that the body mass percentage which maximizes mechanical power was 95.7 ± 10.4% (CV = 10.86%) and 106.4 ± 10.9% (CV = 10.25%) for the BSQ and HBD exercises, respectively. Despite the moderate between-subjects’ variability of this approach, no statistically significant differences were found in power output across a wide range of percentage of body mass for both the BSQ (˜65–123% of body mass) and HBD (˜80–132% of body mass) exercises. Thus, although a more objective assessment is necessary, this approach might be a much more feasible and practical alternative for strength and conditioning coaches to prescribe exercise load when such devices are not available [38].

Despite the novelty of our findings, some study limitations are worth mentioning. Our participants were resistance-trained (population who can lift ˜1.5 [BSQ] and ˜1.8 [HBD] their body mass), which limits the extrapolation of our results to individuals with higher RT backgrounds (i.e., highly trained athletes). Furthermore, we only recruited men, and future studies should investigate the load-velocity and load-power relationships for women; thus, cross-validation of our equations to other populations is required. We only used a linear position encoder to measure bar kinematics, and it would be relevant to use a motion analysis system and muscle activation to provide further understanding of the mechanical pattern and muscle activation in lifting between the exercises performed here.

From a practical perspective, the use of the load-velocity relationships provided here might be useful for strength and conditioning coaches to monitor and prescribe the exercise load of their practitioners in real time on an individual daily basis. For example, a target MPV might be chosen beforehand during the warm-up, depending on the specific training goal being pursued. Furthermore, the range of light-moderate loads that elicited similar power output to P_max_ raises some questions about the effectiveness of determining a single “optimal load”. Collectively, these data might assist strength and conditioning coaches to implement movement velocity as a feasible approach to determine exercise loads. Finally, if devices such as linear encoders are not available, the percentage of body mass could be used as a practical alternative for prescribing exercise load, despite moderate inter-subject variability.

## CONCLUSIONS

In summary, we found that the general load-velocity relationship was highly linear in both the BSQ and HBD exercises, which enables the accurate estimation of the 1RM from MPV recorded during a single repetition performed at maximum effort. In addition, HBD elicited higher mechanical outputs than BSQ, but the range of submaximal loads capable of maximizing power output in both exercises occurred at a quite similar %1RM. Therefore, strength and conditioning coaches can use the load-velocity and load-power relationships provided here as a quick and easy approach to prescribe and adjust the exercise load during RT programmes on a daily basis.
